# Deep Temporal Clustering of Pathological Gait Recovery Patterns in Post-Stroke Patients Using Joint-Angle Trajectories: A Longitudinal Study

**DOI:** 10.3390/bioengineering12121314

**Published:** 2025-11-30

**Authors:** Jinwoo Kim, Teh-Hao Teng, Yun-Hee Kim, Seung-Jong Kim, Mun-Taek Choi

**Affiliations:** 1 Department of Intelligent Robotics, Sungkyunkwan University, Suwon 16419, Republic of Korea; robotjinu25@skku.edu (J.K.); deohao23@skku.edu (T.-H.T.); 2 Department of Physical and Rehabilitation Medicine, Sungkyunkwan University School of Medicine, Suwon 16419, Republic of Korea; yunkim@skku.edu; 3 Myongji Choonhey Rehabilitation Hospital, Seoul 07378, Republic of Korea; 4 Department of Biomedical Engineering, College of Medicine, Korea University, Seoul 02841, Republic of Korea

**Keywords:** post-stroke rehabilitation, longitudinal gait analysis, joint-angle trajectories, deep temporal clustering, time-series data augmentation

## Abstract

This study aims to analyze long-term gait recovery patterns in sub-acute post-stroke hemiplegic patients by applying end-to-end deep learning (DL)-based clustering to sagittal joint-angle trajectories throughout the gait cycle. To address the data scarcity issue in long-term follow-up patient gait trajectory datasets, two time-series data augmentation methods, TimeVAE and Diffusion-TS, were employed to generate high-fidelity synthetic joint-angle trajectories. The augmented dataset were subsequently analyzed using a Deep Temporal Clustering (DTC) model, which effectively captured individualized longitudinal recovery patterns by jointly learning temporal representations and cluster assignments. Based on the clustering evaluation criteria, the model identified six clusters as the optimal grouping. These clusters were statistically well represented by distinct kinematic characteristics. This study represents the first attempt to analyze longitudinal gait recovery patterns in post-stroke patients using a deep clustering model. While exploratory in nature, it provides a conceptual basis for future longitudinal research in stroke rehabilitation.

## 1. Introduction

Stroke is a leading cause of long-term disability, often resulting in hemiplegia that impairs gait due to muscular weakness and neuromuscular dysfunction, particularly in the lower limbs [1]. Although many stroke survivors regain partial motor function, residual gait abnormalities frequently persist, reducing mobility and quality of life [2]. For customized rehabilitation of post-stroke patients with hemiplegic gait, therefore, a systematic analysis of the long-term recovery patterns of hemiplegic gait function is necessary.

With the advent of wearable sensing technologies and machine learning (ML), more data-driven clustering analyses of gait characteristics have become feasible. Chantraine et al. [3] employed k-Means clustering on selected knee kinematic parameters to identify subgroups of stiff-knee gait (SKG) severity, providing a more flexible, data-driven alternative to traditional statistical models. Mulroy et al. [4] applied non-hierarchical clustering to temporal and kinematic gait parameters in post-stroke patients and identified four distinct gait groups. Watari et al. [5] explored gait heterogeneity in runners with patellofemoral pain by applying hierarchical cluster analysis to pelvic acceleration time-series data. Sánchez et al. [6] applied a sparse k-Means clustering approach to a set of spatiotemporal and ground reaction force variables obtained during walking, enabling the data-driven identification of heterogeneous clusters of walking behavior in individuals with chronic stroke and neurotypical controls.

Early machine learning-based methods rely on manually designed gait features, which often have limitations such as bias, lack of consistency, and degraded performance when capturing complex gait dynamics [7]. Deep learning (DL) methods have been introduced to overcome these constraints in gait analysis [8]. Konz et al. [9] incorporated spatiotemporal perspectives into gait clustering, expanding the analytical scope beyond static kinematic parameters and setting the stage for more dynamic modeling approaches. Kim et al. [10] proposed the Simultaneous Clustering and Classification (SCC) framework, which combines deep representation learning with unsupervised clustering to directly process multivariate time-series kinematic data, enabling the model to capture inter-joint coordination and temporal dependencies that conventional feature-based ML approaches could not represent. Taha et al. [11] extended these ideas by developing a deep feature learning and clustering-based framework using inertial measurement unit (IMU) data for gait biometrics, demonstrating the potential of DL for unsupervised gait pattern discovery. Kim et al. [12] applied Deep Temporal Clustering to pathological gait trajectories in a cross-sectional study, highlighting the feasibility of DL-based clustering.

Collectively, these approaches remain largely cross-sectional and provide only static snapshots of post-stroke recovery. Such approaches often fail to capture the dynamic and individualized nature of gait restoration and remain limited in both temporal resolution and generalizability [13]. Therefore, continuous and longitudinal assessments of gait recovery are essential for understanding individual rehabilitation trajectories and developing personalized treatment strategies [14].

Collecting gait measurements at different time points into a coherent longitudinal dataset introduces a data-scarcity challenge, a well-known limitation in biomedical time-series modeling [15]. Various augmentation methods for time-series data have gained attention to mitigate this limitation. Prior studies have shown that appropriate augmentations preserve the underlying data structure. He et al. [16] demonstrated that two augmented views derived from the same instance should correspond to the same representation, reinforcing the principle that augmentation should maintain instance identity rather than distort it. Lu et al. [17] categorize data augmentation invariance as one of the core inductive priors in deep clustering methods, arguing that carefully designed perturbations of the same instance lead to invariant representations and thereby help uncover the latent structural patterns underlying the data. Consequently, data augmentation serves as an effective means to alleviate data scarcity and improve representation robustness.

The objectives of this study is to establish a comprehensive understanding of long-term gait recovery patterns in the rehabilitation of post-stroke hemiplegic patients. First, we aim to identify and characterize the longitudinal patterns of gait recovery by clustering temporal changes in gait features, thereby uncovering representative recovery patterns across individuals. Second, we enhance clustering performance by reducing reliance on human intervention and introducing longitudinal deep learning-based clustering that autonomously extracts meaningful progression patterns from high-dimensional gait time series in an end-to-end manner. Third, considering the scarcity and incompleteness of the long-term dataset, we try to enhance the robustness of clustering performance and improve model generalizability by incorporating deep learning-based missing-data imputation and data augmentation model techniques. Lastly, we provide useful supplementary tools to rehabilitation clinicians by presenting statistical analyses of gait characteristics associated with optimized recovery clusters.

## 2. Related Work

This section provides an overview of the DL models employed in this study, focusing on three key aspects: imputation, data augmentation, and clustering. Imputation models were employed to reconstruct missing values in multivariate time-series data, ensuring temporal continuity and reducing information loss caused by incomplete observations. Data augmentation models were employed to address data scarcity by generating high-fidelity synthetic signals that capture the underlying temporal and longitudinal recovery patterns of gait. Finally, a clustering model was employed to identify latent structures and group-level patterns within the data, facilitating interpretable analyses of complex rehabilitation patterns. Each of these components is described in detail in the following subsections.

### 2.1. Time-Series Data Imputation

**Figure 1:**
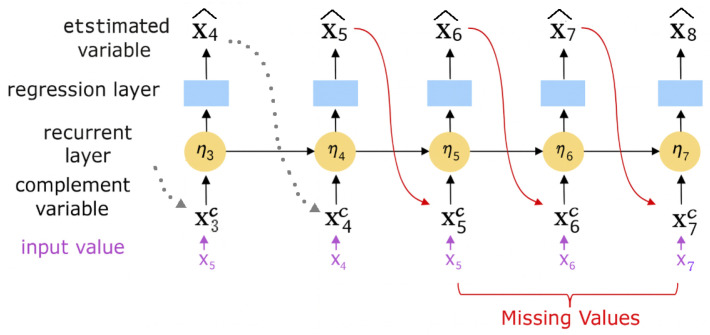
Schematic overview of the BRITS architecture.

BRITS (Bidirectional Recurrent Imputation for Time Series) is a bidirectional RNN framework designed to impute missing values in multivariate time series [18]. The overall architecture of BRITS and its bidirectional imputation mechanism is illustrated in Figure 1. By processing the sequence in both forward and backward directions, BRITS captures underlying temporal dynamics, allowing the model to utilize both past and future contexts. Unlike supervised regression models that require explicit target labels, BRITS adopts a self-supervised learning scheme in which supervision is derived directly from the data itself. In this setting, the supervised labels are simply the observed values themselves in the time series rather than externally provided class annotations or manually defined targets.

During training, a portion of the observed entries is intentionally masked and treated as missing, and the model learns to reconstruct these masked values using the surrounding temporal context. To maintain temporal continuity in the presence of missing inputs, BRITS introduces a complement variable that merges observed and estimated values:

(1) $$X_{t}^{c}=M_{t}⊙X_{t}+\left(1-M_{t}\right)⊙{\hat{X}}_{t},$$

where $M_{t}\in \left\{0,1\right\}$ indicates whether $X_{t}$ is observed ($M_{t}=1$) or missing ($M_{t}=0$). This allows the recurrent layer to receive valid inputs at every step, ensuring that information flows smoothly over time.

Each hidden state $\eta_{t-1}$ encodes temporal information from previous steps and is used by a regression head to estimate the next value:

(2) $${\hat{X}}_{t}=W_{X}\eta_{t-1}+b_{X},$$

where $W_{X}$ and $b_{X}$ are regression parameters. Although this mapping is linear, the hidden state $\eta_{t-1}$ evolves through the recurrent network and captures the underlying temporal dynamics. Thus, the regression head functions purely as a read-out layer, while the nonlinear, context-dependent estimation is driven primarily by the dynamics encoded in the hidden state.

Finally, BRITS minimizes the reconstruction loss between the observed and predicted values:

(3) $$L_{\mathrm{rec}}=\sum_{t}M_{t}⊙{∥X_{t}-{\hat{X}}_{t}∥}_{2}^{2}.$$

Because this loss is computed only over observed entries, the network learns temporal dependencies and missing-value patterns without any external labels, effectively teaching itself how to infer missing data.

### 2.2. Time-Series Data Augmentation

**Figure 2:**
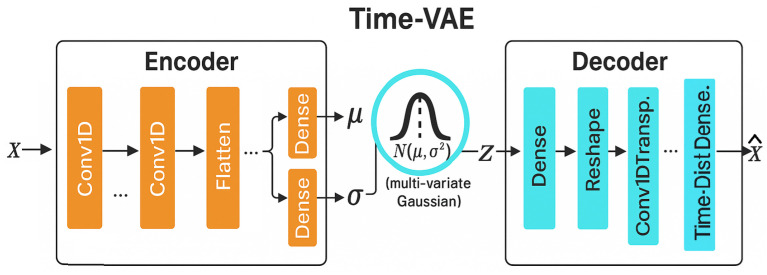
Schematic overview of the TimeVAE architecture.

TimeVAE extends the standard variational autoencoder (VAE) framework to time-series data by explicitly modeling temporal dependencies. As shown in Figure 2, the model consists of an encoder, a latent sampling layer, and a decoder, forming a generative pipeline that learns to reconstruct and generate sequential signals.

The encoder transforms each input sequence $X\in R^{T\times d}$ into two latent parameters, the mean μ and standard deviation σ, which define a multivariate Gaussian distribution. A latent vector *z* is then sampled using the reparameterization trick:

(4) z=μ+σ⊙ϵ,ϵ∼N(0,I),

where random noise ϵ enables gradient flow through the stochastic sampling process. This latent representation compresses temporal patterns while preserving both short- and long-term dependencies. The decoder then reconstructs the original sequence $\hat{X}$ from *z* through a series of deconvolutional (Conv1DTranspose) and time-distributed layers. By sampling new latent vectors from the learned latent space, TimeVAE can generate realistic and diverse time-series trajectories that maintain temporal smoothness and inter-variable consistency.

**Figure 3:**
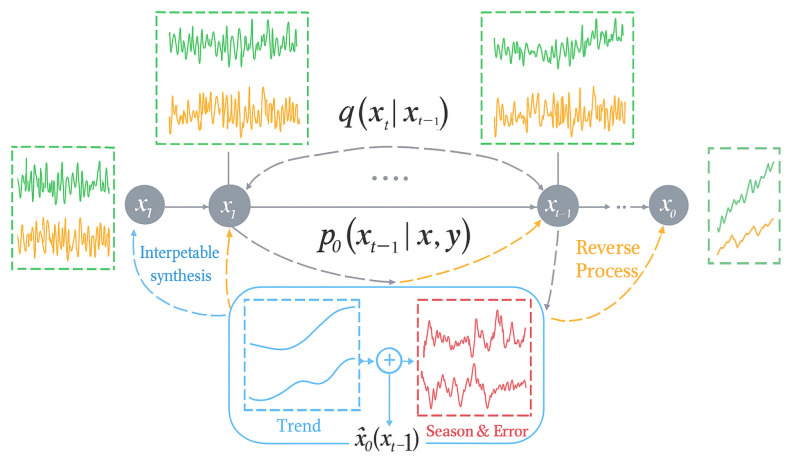
Schematic overview of the Diffusion-TS architecture.

Diffusion-TS is a generative model built upon the denoising diffusion probabilistic model (DDPM), which has recently demonstrated remarkable performance in synthesizing realistic time-series data [19]. As illustrated in Figure 3, the model operates in two complementary stages: a forward diffusion stage that progressively corrupts the input signal by adding Gaussian noise and a reverse denoising stage that reconstructs coherent and realistic trajectories from noisy representations. Through this bidirectional process, Diffusion-TS learns to generate interpretable and high-fidelity time-series data that preserve both long-term patterns and fine-grained temporal variations.

In the forward process, a clean sequence $x_{0}$ is progressively corrupted into Gaussian noise through *T* steps:

(5) $$q\left(x_{t}|x_{t-1}\right)=N\left(x_{t};\sqrt{1-\beta_{t}}\;x_{t-1},\beta_{t}I\right),$$

where $\beta_{t}$ denotes the noise variance schedule controlling the corruption intensity at each step. This stochastic transformation yields a set of noisy samples ${\left\{x_{t}\right\}}_{t=1}^{T}$ that encode temporal uncertainty. During the reverse process, a neural network $\epsilon_{\theta }\left(x_{t},t\right)$ learns to recover the clean signal by estimating the mean $\mu_{\theta }\left(x_{t},t\right)$ and covariance $\Sigma_{\theta }\left(x_{t},t\right)$ of the conditional distribution:

(6) $$p_{\theta }\left(x_{t-1}|x_{t}\right)=N\left(x_{t-1};\mu_{\theta }\left(x_{t},t\right),\Sigma_{\theta }\left(x_{t},t\right)\right).$$

Starting from pure noise $x_{T}∼N\left(0,I\right)$, the model iteratively denoises each step to reconstruct $x_{0}$. Through this reverse sampling, Diffusion-TS synthesizes interpretable time-series data where long-term trends and short-term fluctuations are both realistically preserved.

### 2.3. Time-Series Clustering

**Figure 4:**
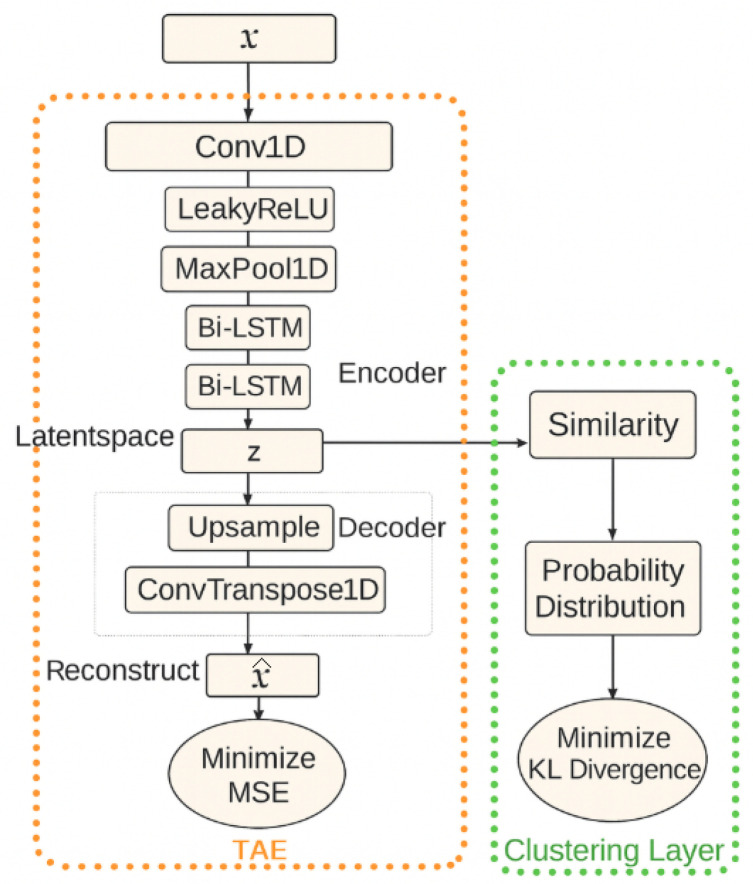
Schematic overview of DTC architecture.

Deep Temporal Clustering (DTC) is a fully unsupervised learning algorithm specifically designed for clustering complex time-series data [20]. As shown in Figure 4, the DTC model integrates a temporal autoencoder with a clustering layer.

The model first encodes multivariate time-series data into a latent representation and subsequently clusters similar latent embeddings based on their temporal similarity [21]. The encoder is responsible for compressing high-dimensional time-series input $X\in R^{T\times d}$ (where *T* is the time length and *d* is the number of features) into a more compact latent representation $Z\in R^{T\times z}$, where *z* denotes the latent variable of the encoder:

(7) $$Z=f_{\mathrm{enc}}\left(X,\theta_{\mathrm{enc}}\right)$$

The model architecture combines one-dimensional convolutional neural networks (1D-CNNs) with Leaky ReLU activations and bidirectional long short-term memory (Bi-LSTM) networks. Each convolutional block in the encoder consists of a Conv1D layer followed by a Leaky ReLU activation function, which extracts spatial dependencies across features. A subsequent max-pooling operation reduces dimensionality while retaining the most salient features. The resulting representations are then passed to Bi-LSTM networks, enabling the model to learn long-range temporal dynamics and richer time-series representations. This design allows the DTC model to effectively capture distinct temporal features without manual feature engineering [7].

Once the input is encoded into the latent space, it is fed into the clustering module. Temporal clustering is performed on this latent representation using a metric-based clustering approach. The clustering layer works by initializing cluster centroids $\mu_{j}$ using hierarchical clustering, where j∈{1,2,…,K}, and *K* is the number of clusters. The soft assignment of each latent point to a cluster $q_{ij}$ is computed using a Student’s t-distribution [22]:

(8) $$q_{ij}=\frac{\left(1+|\right|Z_{i}-\mu_{j}{\left|\right|}^{2}{)}^{-1}}{\sum_{j^{\prime }}\left(1+|\right|Z_{i}-\mu_{j^{\prime }}{\left|\right|}^{2}{)}^{-1}}$$

This soft clustering method allows for smooth transitions between different clusters, ensuring that slight variations in time-series data are captured. To ensure that the latent representation retains critical information about the original input data, the DTC model includes a decoder that reconstructs the time series from the latent representation.

(9) $$\hat{X}=f_{\mathrm{dec}}\left(Z,\theta_{\mathrm{dec}}\right)$$

The decoder consists of an up-sampling layer followed by a deconvolutional layer to reconstruct the input data into reconstructed output $\hat{X}$, as given in Equation (9). This design ensures that the decoded latent space accurately reflects the original time-series data. Notably, reconstruction itself is not an end goal in DTC; rather, it encourages the encoder to learn informative representations that capture temporal dynamics and support multivariate time-series clustering.

Finally, the DTC model is optimized using a composite loss function that integrates both reconstruction and clustering losses. The reconstruction loss is the mean squared error (MSE) between *X* and $\hat{X}$:

(10) $$L_{\mathrm{rec}}=||X-\hat{X}{\left|\right|}^{2}$$

Clustering loss is based on the Kullback–Leibler (KL) divergence, which ensures that the soft assignments $q_{ij}$ closely align with the true clustering distribution $p_{ij}$:

(11) $$L_{\mathrm{clus}}=\sum_{i=1}^{n}\sum_{j=1}^{k}p_{ij}\log\frac{p_{ij}}{q_{ij}}$$

The total cost function $L_{\mathrm{total}}$ that drives end-to-end optimization is given by the sum of the clustering loss and the reconstruction loss:

(12) $$L_{\mathrm{total}}=L_{\mathrm{rec}}+L_{\mathrm{clus}}$$

Through this end-to-end joint optimization, the encoder, decoder, and clustering parameters are simultaneously updated, enabling the model to learn cluster-discriminative latent features while preserving temporal fidelity in reconstruction and dynamically adapting the cluster centroids for unsupervised learning.

## 3. Materials and Methods

**Figure 5:**
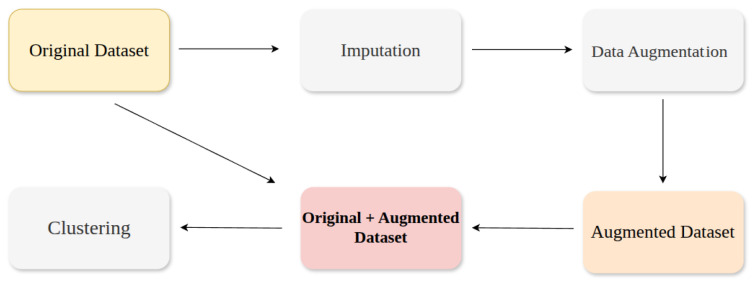
Overall analysis pipeline consisting of data imputation, augmentation, and clustering procedures.

In this study, the entire experimental process followed a structured and sequential analysis pipeline consisting of data collection, preprocessing with missing value imputation, data augmentation, clustering, and statistical analysis, as illustrated in Figure 5. The objective was to establish a reproducible and reliable workflow for analyzing gait recovery trajectories using both real and augmented data.

The first step involved preprocessing gait data, which contained joint-angle trajectories recorded across gait cycles. Because such biomechanical data are often noisy and incomplete, careful preprocessing was necessary to prevent bias in subsequent analysis. A moving average filter was initially applied to smooth the trajectories while preserving key gait dynamics. Despite this smoothing process, missing values remained a major challenge because the time-series augmentation models used later (TimeVAE and Diffusion-TS) cannot be trained directly on sequences containing missing observations, necessitating a complete imputation step prior to model training. Simple imputation methods, such as mean filling, logarithmic interpolation, or neighboring-week filling, were unsuitable because human joint-angle trajectories exhibit complex temporal patterns that cannot be captured through basic interpolation or filling methods.

**Figure 6:**
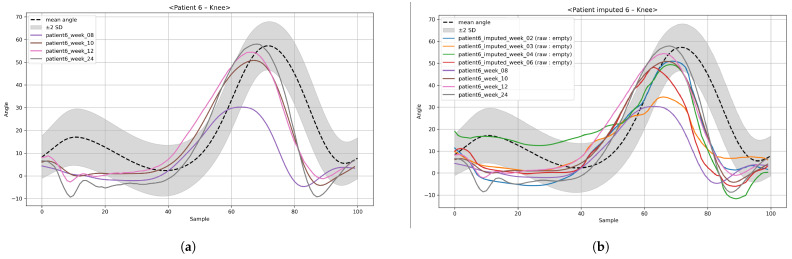
Time-series imputation for joint-angle trajectories: (**a**) raw trajectories and (**b**) BRITS-imputed trajectories reconstructed for missing weeks.

To address this, we employed BRITS, a recurrent neural network (RNN)-based imputation model widely recognized as a benchmark in time-series data reconstruction. Figure 6 compares the raw trajectories with the BRITS-imputed results, showing that the imputed data accurately capture realistic gait motion patterns without oversmoothing. Hyperparameters for BRITS were selected through a grid search to minimize the reconstruction loss.

**Figure 7:**
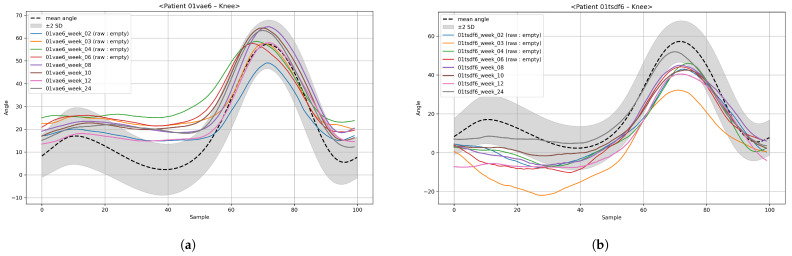
Comparison of time-series augmentation for knee-angle trajectories for a patient: (**a**) augmented TimeVAE and (**b**) augmented Diffusion-TS.

After imputation, data augmentation was applied to increase the diversity of training samples and improve model generalization. Two state-of-the-art time-series generative models were adopted: TimeVAE and Diffusion-TS. These models are capable of capturing complex temporal dependencies and generating realistic synthetic trajectories that maintain the underlying biomechanical properties of gait. Figure 7 illustrates examples of trajectories generated from BRITS-imputed data using TimeVAE and Diffusion-TS, demonstrating that both models successfully preserve the essential temporal features of the knee’s motion.

Previous studies have explored different augmentation ratios to evaluate how additional samples affect learning stability. Guo et al. [23] tested ratios of 1, 2, 5, 10, and 100 for EEG-based seizure detection, while Seyfi et al. [24] used ratios of 2, 4, 6, 8, and 10 for multivariate sensor data. Based on these prior findings, an augmentation ratio of five was selected in this study to ensure a balance between computational efficiency and stable performance in downstream clustering. By incorporating the augmented trajectories alongside the original observations, we mitigated artifacts that might otherwise arise from naive masking or simple interpolation, thereby preserving both short-term fluctuations and long-term trends [25]. As with BRITS, the hyperparameters for both TimeVAE and Diffusion-TS were tuned through a grid search to optimize each model’s respective loss function.

Finally, clustering was performed using the DTC model, which integrates a temporal autoencoder (TAE) and a clustering layer in an end-to-end framework. To ensure optimal clustering performance, hyperparameter optimization was carried out using Optuna (version 4.4.0) [26], a Bayesian optimization-based framework that efficiently explores hyperparameter spaces. Following the approach of Batool et al. [27], who optimized clustering using the Silhouette function, this study employed the Balanced Silhouette Score (BSS) as the objective function in Optuna, as further described in Section 3.4.

**Figure 8:**
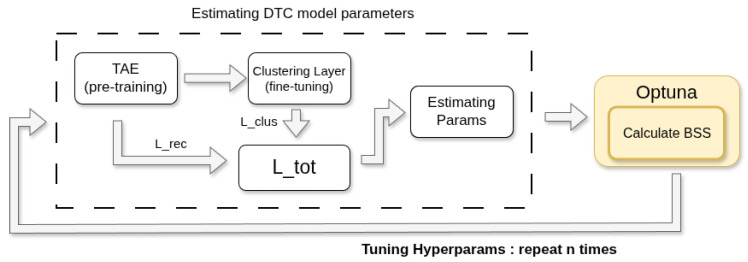
Iterative hyperparameter optimization process for DTC using the Balanced Silhouette Score (BSS) as the objective function.

As illustrated in Figure 8, for each predefined number of clusters *K*, the TAE and clustering module were jointly trained by minimizing a total loss function $L_{\mathrm{total}}$, as described in Section 2.3. For each *K*, the configuration with hyperparameters—such as learning rate and pool size—that were automatically tuned across multiple trials and achieved the highest BSS after *n* iterations was selected as the optimal model for subsequent analyses.

In the following, Section 3.1, Section 3.2, Section 3.3 and Section 3.4 elaborate on the methodological framework and provide detailed explanations of each analytical stage. Specifically, they outline data-collection procedures, the evaluation of imputation and data-augmentation effectiveness, the metrics used to assess clustering performance, and the methods employed to validate the resulting optimal clusters.

### 3.1. Data Collection

**Figure 9:**
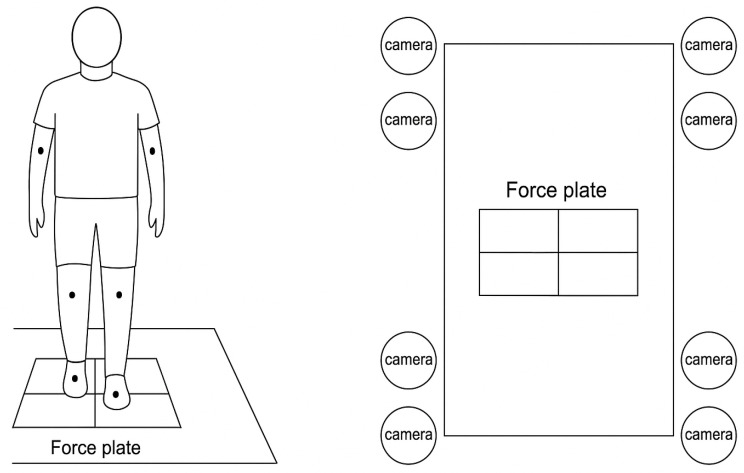
Motion capture system with the Helen–Hayes marker set.

This study involved 31 sub-acute hemiplegic post-stroke patients, with a primary focus on analyzing lower limb movements, particularly in hip, knee, and ankle joints. Gait assessments for the patients were conducted at 2, 3, 4, 6, 8, 10, 12, and 24 weeks after onset to monitor gait recovery [28]. As illustrated in Figure 9, the patients performed the tests walking on a 10-meter track, with a force plate placed in the center, while cameras positioned both in front and behind the track recorded the markers’ movements. This setup allowed for the precise tracking of lower limb kinematics, including spatiotemporal, kinematic, and clinical assessment data.

Data collection was carried out from May 2017 to August 2022 at the Samsung Medical Center (SMC) in South Korea, following approval by the Institutional Review Board (IRB approval number: SMC 2017-11-081) and obtaining informed consent from all participants. Gait data were obtained across eight follow-up periods after stroke onset. During a six-month observation period, each patient’s gait data were gathered using the Orthotrak motion capture system (version 6.6.4) operating at a 60 Hz sample rate [29]. This system captured joint angles and angular velocities, focusing on joint-level kinematic details at the hip, knee, and ankle [30]. Markers were positioned following the Helen–Hayes marker set guidelines [31].

Table 1 presents a sample of 10 subjects, summarizing key clinical, stroke-related, and spatiotemporal gait features. During the early rehabilitation phase, several patients were unable to complete independent gait cycles, resulting in incomplete trajectory segments. Additional data gaps occurred due to mid-study discharge, voluntary withdrawal, or missed assessment sessions, leading to missing measurements at specific follow-up points. As a result, missing values were unavoidable.

Table 2 summarizes the distribution of patients according to the number of weeks for which gait data were recorded. For example, five patients had six measured weeks, resulting in a total of 30 gait instances for that group. To ensure data reliability, only patients with at least four recorded weekly assessments were included in the analysis, resulting in a final cohort of 31 patients. If all participants had completed all eight scheduled assessments, the dataset would comprise 248 patient-weeks (31 patients × 8 weeks). In practice, 59 of these entries were missing, corresponding to an overall missing rate of approximately 24%.

### 3.2. Dataset

The datasets used in this study as primary inputs to the DTC are joint-angle trajectories during gaits of post-stroke hemiplegic patients. The analysis focused on sagittal-plane joint-angle trajectories, as they capture the essential biomechanical components of walking—especially hip, knee, and ankle flexion–extension patterns that contribute to propulsion and balance [32]. While the coronal and transverse planes contribute to coordination and stability, they provide less direct information for modeling gait dynamics. Focusing on the sagittal plane therefore enables efficient and interpretable clustering outcomes without loss of clinically relevant information [32].

Following data collection, sagittal-plane joint-angle and angular velocity trajectories of the hip, knee, and ankle were time-normalized to 100 points per gait cycle, resulting in a total of 600 time steps. This yielded 248 samples (31 patients × 8 weeks), each originally stored as an independent one-dimensional time-series sequence of length 600, with unrecorded trajectories left vacant. During tensor [33] construction, the weekly trajectories belonging to the same patient—originally stored as independent rows—were grouped together and stacked in chronological order. Consequently, each patient was represented as a single multivariate time-series sample of size 8×600 (or equivalently 1×8×600), corresponding to eight time-series sequences per individual. The full dataset was therefore structured as a three-dimensional tensor of shape [31,8,600], providing a consistent subject-wise temporal organization. Although the DTC model operates on two-dimensional multivariate time-series inputs (i.e., T×F), it processes each patient’s sequence individually in this 8×600 format. The [31,8,600] tensor simply adds a batch dimension, allowing multiple patient sequences to be passed through the DTC model simultaneously during training.

### 3.3. Augmentation Assessment

To evaluate the quality of the generated trajectories, both quantitative and qualitative assessments were conducted. For the quantitative evaluation, the Maximum Mean Discrepancy (MMD) was employed to measure the distributional difference between the original and augmented trajectory datasets [34]. MMD quantifies the distance between two probability distributions *P* and *Q* in a reproducing kernel Hilbert space (RKHS):

(13) $${\mathrm{MMD}}^{2}\left(P,Q\right)=E_{x,x^{\prime }∼P}\left[k\left(x,x^{\prime }\right)\right]+E_{y,y^{\prime }∼Q}\left[k\left(y,y^{\prime }\right)\right]-2E_{x∼P,y∼Q}\left[k\left(x,y\right)\right],$$

where k(·,·) denotes a positive-definite kernel function. In this study, a Gaussian RBF kernel was used, $k\left(x,y\right)=\exp(-∥x-y{∥}^{2}/2\sigma^{2})$, and the bandwidth σ was determined using the median heuristic, following Sutherland et al. [35]. When the two distributions are identical, MMD approaches zero, while larger values indicate greater divergence.

To assess whether the augmented trajectories preserved the same underlying distribution as the original data, a permutation test was conducted to evaluate the statistical significance of the observed MMD value [36]. Specifically, the p-value was estimated following the permutation-based procedure described by Gretton et al. [37], incorporating the plus-one correction proposed by Phipson and Smyth [38] to ensure robustness under finite sampling. In this approach, an empirical null distribution of MMD statistics is generated through repeated random label shuffling, and the statistical significance of the observed MMD between the original and augmented datasets is evaluated accordingly.

For the qualitative evaluation, we employed the Uniform Manifold Approximation and Projection (UMAP) [39] to visualize high-dimensional data in a low-dimensional space and compare the structural organization of the original and augmented trajectories. UMAP has become a widely used tool for visualization in time-series research. It is particularly effective for projecting latent representations learned by deep sequence models such as RNN- and Transformer-based architectures [40] and for inspecting latent spaces in time-series anomaly detection and forecasting tasks [41]. UMAP computes low-dimensional coordinates by minimizing the cross-entropy between data points, providing a visualization of high-dimensional data that preserves local neighborhood relationships and the nonlinear geometric structure of the original manifold to the greatest extent possible. The resulting low-dimensional coordinates (typically 2D or 3D) provide a faithful embedding of these nonlinear structures, enabling the qualitative assessment of structural similarity and manifold consistency between the original and augmented datasets while maintaining temporal continuity.

### 3.4. Clustering Assessment

Clustering assessment in this study consists of two complementary stages:

(1) Determining the optimal number of clusters;
(2) Evaluating the statistical validity of the resulting cluster assignments once the optimal number of clusters has been selected.

As the first stage of determining optimal number of clusters, the Silhouette Coefficient (SC) [42] is used to quantify the cohesion and separation of each resultant cluster. This internal validation metric is particularly valuable in unsupervised learning settings, where no ground-truth labels are available, because it assesses clustering quality by jointly evaluating intra-cluster cohesion and inter-cluster separation. Specifically, the SC evaluates how tightly grouped a data point is within its own cluster and how distinctly it is separated from other clusters. The SC is defined on a scale from −1 to 1, where values close to 1 indicate that the data points are well-clustered, with high within-cluster density and clear separation from other clusters [42]. A score near 0 suggests overlapping cluster boundaries, and negative values imply the misclassification of data points, where a point is likely assigned to the wrong cluster. The SC for each data point *i* is computed based on two key distances:

- a(i): The mean intra-cluster distance or the average distance between point *i* and all other points in the same cluster.
- b(i): The mean nearest-cluster distance or the average distance between point *i* and points in the closest neighboring cluster.

These distances are combined to calculate the silhouette score:

(14) $$SC\left(i\right)=\frac{b(i)-a(i)}{\max(a(i),b(i))}$$

Nevertheless, although the SC provides a meaningful evaluation of clustering quality when the number of clusters *K* is fixed, it does not directly quantify how well different choices of *K* partition the data as a whole. In other words, the SC can compare the relative quality of clusters produced under a predefined *K*, but an additional measure is required when the goal is to determine which value of *K* yields the most coherent global structure. For this reason, we employ the Silhouette Score (SS) [42], which aggregates SC values across all samples to provide a unified criterion for selecting the optimal number of clusters. The SS is computed as:

(15) $$SS=\frac{1}{n}\sum_{i=1}^{n}SC\left(i\right)$$

While the metric effectively captures cluster compactness and separability, optimizing it in isolation systematically biases the solution toward dominant clusters as the dataset size grows, as noted by Lorenz et al [43]. In the context of longitudinal gait trajectory analysis, this led to the excessive aggregation of samples into a single large cluster, thereby reducing the practical interpretability of the results.

To address this limitation, an entropy term (*H*) was incorporated to quantify the uniformity of the cluster membership distribution, drawing inspiration from the entropy-based clustering assessment described by Dong et al. [44]. Specifically, Shannon’s entropy [45], a measure derived from information theory, is computed as:

(16) $$H=-\sum_{j=1}^{K}p_{j}\log\left(p_{j}\right),\;p_{j}=\frac{k_{j}}{N}$$

where $k_{j}$ is the number of samples in cluster *j*, and *N* is the total number of samples. This measure penalizes cluster imbalance by increasing when data points are evenly distributed across clusters and decreasing when one cluster dominates.

By combining SS and entropy into a normalized term whose range is confined to [0, 1], a balanced evaluation index—termed the Balanced Silhouette Score (BSS)—was introduced to jointly account for clustering quality and cluster-size uniformity:

(17) $$BSS=\left(1-\alpha \right)\cdot SS_{\mathrm{normalized}}+\alpha \cdot H_{\mathrm{normalized}}$$

This balanced metric mitigated the over-concentration of data into a single cluster and facilitated a more meaningful interpretation of patterns in longitudinal dataset. Here, α denotes a weighting coefficient that controls the trade-off between clustering quality and entropy-based balance. Setting α too high leads to excessively large entropy values during optimization, resulting in overly diffuse assignments and reduced silhouette scores, whereas setting it too low leads to overly concentrated assignments in a single cluster, reducing the meaningful separation between groups. Based on the trade-off between entropy regularization and cluster separation, α=0.15 was set a priori as a predefined hyperparameter rather than being optimized during training.

After determining the optimal number of clusters, a statistical validation procedure was conducted to assess whether the identified groups exhibited meaningful and temporally consistent differences. Because repeated-measures frameworks are widely used in longitudinal analysis to evaluate within-subject changes over time [46], two-way mixed ANOVA [47] was employed for this purpose. This mixed-design structure differs from conventional two-way ANOVA in that the latter treats all factors as between-subject variables and therefore cannot accommodate repeated measurements from the same individuals over time. In the mixed-design ANOVA used in this study, the group factor was treated as a between-subject variable, whereas the week factor was treated as a within-subject (repeated-measures) variable. By incorporating both between- and within-subject effects, the mixed ANOVA enables simultaneous evaluation of group–time interactions, thereby providing a rigorous assessment of whether the temporal evolution of kinematic trajectories differs meaningfully across the identified groups.

## 4. Results

### 4.1. Data Augmentation

In this study, two augmentation methods—TimeVAE and DiffusionTS—were applied. To assess whether the augmented samples preserved the original data distribution, we tested the null hypothesis stating that the original and augmented datasets are distributionally equivalent using a permutation-based MMD test, as described in Section 3.3. The hyperparameter settings used for TimeVAE are summarized in Table 3. As a result, a *p*-value of 0.069 was obtained for TimeVAE, indicating that the null hypothesis of distributional equivalence could not be rejected at the 0.05 significance level, as summarized in Table 4.

In contrast, generated trajectories by Diffusion-TS showed a statistically significant deviation from the original data (p<0.001), leading to the rejection of the null hypothesis and indicating a substantial distributional shift. This result stands in sharp contrast to TimeVAE, for which its non-significant *p*-value (0.069) suggests a much closer alignment with the original data distribution.

**Figure 10:**
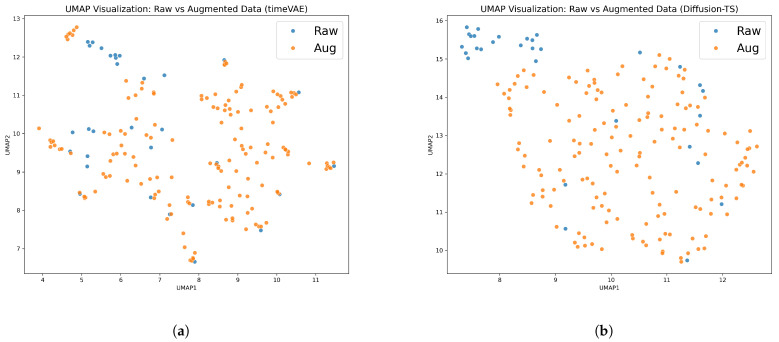
Visualization of raw and augmented data distributions using UMAP: (**a**) TimeVAE-augmented and (**b**) Diffusion-TS-augmented. Colors represent raw (blue) and augmented (orange) samples, with each point representing a single gait trajectory.

Moreover, as shown in Figure 10, the UMAP visualization reinforced these results, as the TimeVAE-augmented data demonstrated a more coherent alignment and natural overlap with the original trajectories, indicating superior preservation of the intrinsic data structure. These results imply that TimeVAE better preserves the intrinsic characteristics of the original trajectories, potentially due to the limited data size constraining the training stability of the diffusion model. Consequently, TimeVAE-generated data were primarily utilized for the clustering analysis.

### 4.2. DTC Clustering

This section presents the results of determining the optimal number of clusters *K*. For each candidate *K* ranging from 3 to 10, hyperparameter optimization was performed using the Bayesian optimization–based library Optuna. A total of 300 iterative trials were conducted for each setting to explore various parameter combinations, including batch size, learning rate, and pool size. The optimal configuration for each *K* was selected based on the highest BSS value obtained across all trials. Table 5 summarizes the hyperparameter search ranges used to determine the optimal K.

As shown in Table 6, the effect of data augmentation on clustering was evaluated by comparing two experimental settings:

- Using only the original dataset;
- Using both the original and augmented datasets.

This comparison allowed us to assess whether augmentation-induced variability enhanced clustering robustness and improved the generalization of trajectory pattern identification. In general, the augmented setting consistently outperformed the original setting across all tested values of *K*, yielding higher BSS scores and exhibiting stable *H* values around 0.8 or higher, thereby validating the intended effect of the entropy-regularization parameter α. Based on these results, the optimal number of clusters was determined to be K=6, as this setting yielded the highest BSS value of 0.780 under the augmented configuration.

Although data augmentation was applied, the overall dataset remains relatively small compared to typical machine learning tasks, making training stability a primary consideration. For this reason, a full-batch strategy was adopted. Using the entire dataset in each update provides variance-free and, therefore, more stable gradient estimates than mini-batch sampling [48]. In the encoder, the two Bi-LSTM layers were assigned fixed hidden sizes of 50 and 1, respectively. These settings follow common DTC practice, as the unsupervised nature of the method makes detailed, task-specific hyperparameter tuning difficult [20]. Detailed information about the group members is presented in Table 7.

### 4.3. Statistical Analysis of Optimal Recovery Clusters

Statistical analysis was performed on commonly used kinematic features, including hip flexion/extension, knee flexion/extension, and ankle dorsiflexion/plantarflexion [5]. Although all peak metrics derivable from the combined original and augmented joint trajectories, we report only three representative measures—peak hip extension, peak knee flexion, and peak ankle dorsiflexion—to ensure concise presentation and maintain analytic clarity.

To further characterize the differences among the optimal groups, a week-specific RMSE analysis was performed by comparing the measured gait trajectories of each cluster with the normal reference trajectories obtained from the control group. RMSE is frequently used in gait and kinematic analyses as a quantitative indicator of trajectory deviation or measurement error in time-series joint-angle or kinematic data [49]. A low week-specific RMSE value indicates that the gait trajectory closely follows the normative pattern for that week, whereas a high RMSE reflects a larger deviation from the control reference in that week, indicating poorer gait function.

For each measurement week(∈{2,3,4,6,8,10,12}), the RMSE was computed separately to quantify the temporal evolution of gait trajectory deviation. The week-specific RMSE was calculated according to Equation (18), where $n_{week}$ denotes the total number of time points in week, $y_{t}$ represents the patient’s joint angle at time point *t*, and ${\hat{y}}_{t}$ denotes the corresponding joint angle from the control group:

(18) $${RMSE}_{\mathrm{week}}=\sqrt{\frac{1}{n_{\mathrm{week}}}\sum_{t\in \mathrm{week}}{\left(y_{t}-{\hat{y}}_{t}\right)}^{2}},$$

**Figure 11:**
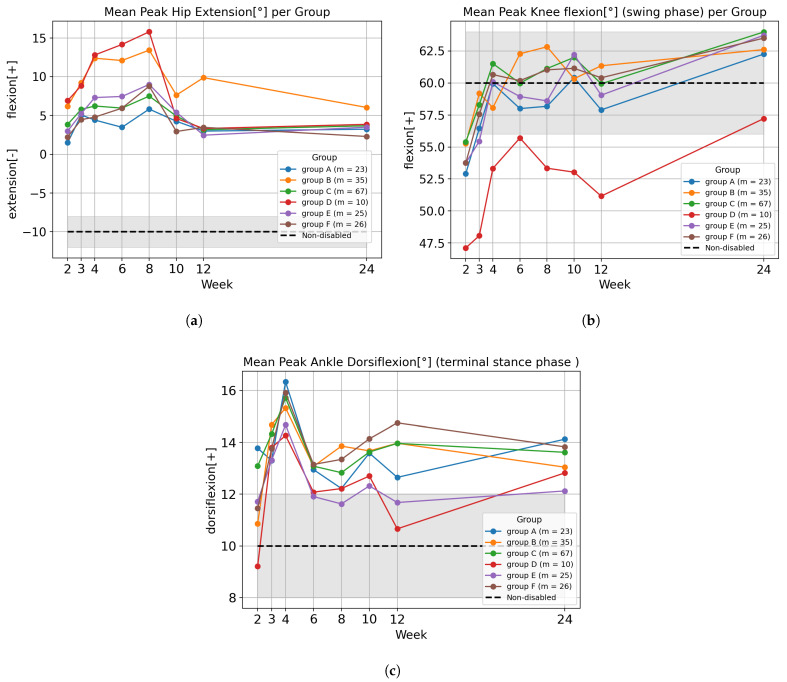
Temporal trends in gait kinematic features for each recovery group. (**a**) Peak Hip Extension; (**b**) Peak Knee Flexion; (**c**) Peak Ankle Dorsiflexion.

**Figure 12:**
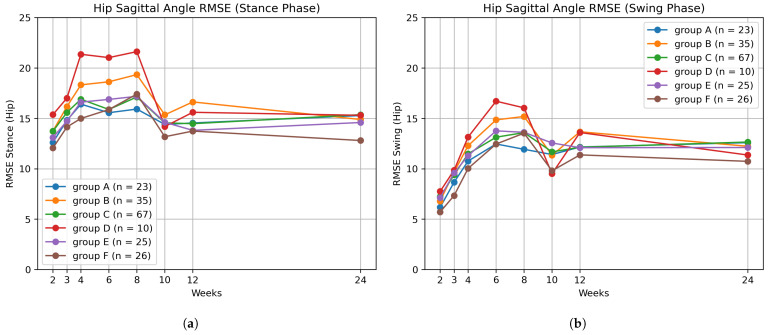
Degree of trajectory deviation compared to non-disabled individuals. (**a**) Stance-phase hip RMSE; (**b**) Swing-phase hip RMSE.

Visualizations of the kinematic features are provided in Figure 11, whereas Figure 12 illustrates RMSE results using a representative example of the hip joint across the stance and swing phases. For RMSE computation, each gait cycle was divided into stance and swing phases, with the stance phase comprising approximately 60% of the cycle and the swing phase comprising the remaining 40%. Each feature was organized by group and by week, and these week- and group-specific values were subsequently used for statistical analysis. To improve visual clarity, only the group averages are shown without standard deviation bands.

To verify whether the optimal clusters were statistically well separated, a two-way mixed ANOVA was conducted. The within-subject factor was defined as week, representing repeated measurements for the same participants across different time points. The between-subject factor was defined as group, since participants belonged exclusively to one cluster, and no repeated measures were involved between groups. The primary objective of this analysis is to examine interaction effect of week and group, which reveals whether the temporal progression differs among groups. This approach provides a statistical validation of the clustering results beyond the deep learning-based evaluation in Section 4.2.

The statistical significance of kinematic features was verified using two-way ANOVA, as shown in Table 8. For the hip joint, all three metrics—peak extension and RMSE in both stance and swing phases—showed significant interaction effects (p<0.01), indicating that hip kinematic patterns evolved differently across groups over time. For the knee joint, the interaction effect was significant only for peak flexion during the swing phase (p<0.05), whereas the RMSE metrics did not reach statistical significance. This suggests that knee flexion amplitude, rather than overall trajectory deviation, was the primary factor distinguishing temporal changes among clusters. For the ankle joint, peak dorsiflexion in the terminal stance phase exhibited a significant interaction effect (p<0.005). Additionally, the ankle RMSE in the swing phase showed a significant interaction (p<0.05), while the stance-phase RMSE did not. These findings indicate that ankle motion during late stance and swing phases contributes most strongly to the temporal differentiation among groups. Taken together, these results confirm that the optimal groups identified by DTC exhibit distinct longitudinal trajectories, with specific kinematic variables showing significantly different temporal patterns across groups.

## 5. Discussion

For the methodological preparation of this exploratory study, an a priori sample size estimation was performed using an independent t-test. This estimation was informed by a longitudinal follow-up study carried out by Kollen et al. [50] on gait recovery after stroke, which reported a mean difference in the gait speed of 0.187 m/s between 1 week and 6 months post-onset, with a standard deviation of 0.3 m/s. Using G*Power [51], a two-tailed test with a significance level of α=0.05 and a desired statistical power of 95% yielded a required sample size of 36 participants. The effect size used for this estimation was derived from the longitudinal findings of Kollen et al. [50], who reported a mean difference in gait speed of 0.187 m/s between 1 week and 6 months post-onset with a standard deviation of 0.3 m/s. In the current dataset, 31 participants were available, which is slightly below the a priori estimate of 36. Although the available sample of 31 participants is modest yet meaningful for exploratory analysis, it remains insufficient for more definitive, large-scale modeling. To compensate for the limited sample size, data augmentation techniques were employed to expand the dataset. Augmentation was not used to generate independent samples [52] but to expose the model to plausible temporal variations and reinforce existing temporal patterns under a small-sample regime [53]. Nonetheless, this time-series augmentation remains meaningful in this exploratory study.

**Figure 13:**
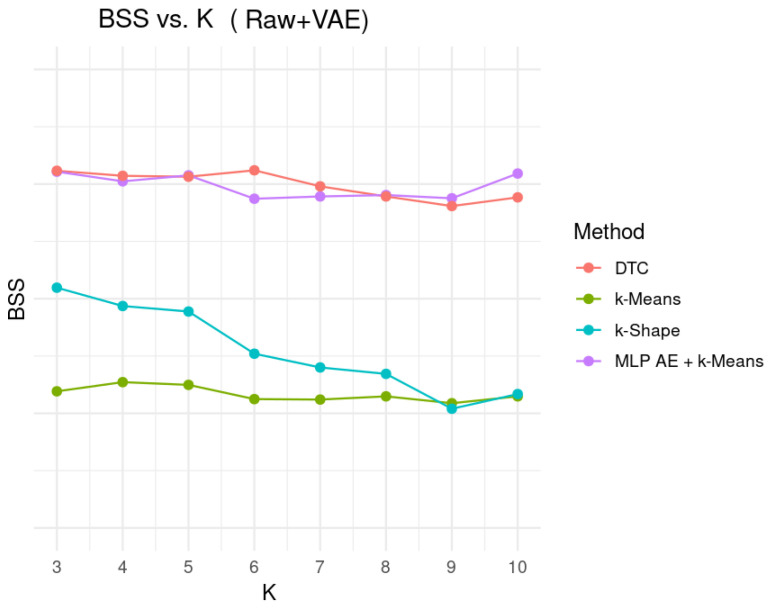
Clustering performance comparison between different algorithms.

Additional comparisons were conducted across multiple clustering models on the augmented dataset, and the DTC method consistently demonstrated the most stable and coherent structure. In this comparative analysis, four clustering approaches were evaluated—k-Means [54], k-Shape [55], MLP Autoencoder + k-Means [56], and DTC—across a range of cluster numbers (K = 3–10). As shown in Figure 13, k-Means produced the lowest BSS across all K with minimal variation, indicating consistently weak separability. In comparison, k-Shape exhibited a gradual decline in BSS as K increased, suggesting reduced separability and greater sensitivity to cluster cardinality. The MLP Autoencoder + k-Means model performed comparably to DTC at small K and showed lower BSS in the mid-range (K = 5–7) while still outperforming the traditional methods such as k-Means and k-Shape by a meaningful margin. DTC maintained the highest and most stable BSS values over all tested K, reflecting superior robustness to cluster-number variation and greater coherence in the underlying temporal representations. These observations further support the selection of DTC as the primary clustering framework in this study.

## 6. Conclusions

This exploratory study established a technical pipeline aiming to comprehensive understanding of long-term gait recovery patterns in the rehabilitation of post-stroke hemiplegic patients. First, we developed a DL-based clustering model that identified the longitudinal patterns of gait recovery by analyzing temporal changes in gait features. Second, we incorporated longitudinal deep learning-based clustering that autonomously extracts meaningful progression patterns from high-dimensional gait time series in an end-to-end manner to enhance clustering performance by reducing reliance on human intervention. Third, we applied deep learning-based missing-data imputation and data augmentation model techniques to enhance the robustness of clustering performance and improve model generalizability. Lastly, we presented the statistical analyses of gait characteristics associated with optimized recovery clusters by providing useful supplementary tools to rehabilitation clinicians.

Future research may further enhance longitudinal gait analysis in post-stroke populations along two complementary directions. The analytical scope can be broadened beyond sagittal-plane kinematics to include frontal- and transverse-plane measures. In addition, it can be extended to incorporate further descriptors such as gait balance indicators and muscle activation profiles. Given the limited amount of longitudinal data currently available, additional data collection is required to enhance the generalizability of the clustering results. Because the current clustering results were obtained through AI-based analytical methods, additional clinical validation is required before the findings can be applied in real-world practice. To develop the proposed approach into a clinically meaningful decision-support tool, it should be integrated within actual rehabilitation workflows, and its contribution to clinical decision-making should be systematically evaluated.

## Figures and Tables

**Table 1:** Clinical data for 10 patients out of the total.

Patient Index	1	2	3	4	5	6	7	8	9	10
Basic Information										
Gender	M	M	M	M	M	M	M	M	M	M
Age (years)	24	25	35	38	45	46	47	58	59	60
Height (cm)	171	178	180	178	180	176	173	168	178	165
BMI	24.28	25.75	25.62	27.77	24.69	24.86	18.74	28.00	22.00	24.98
Alcohol	mod.	mod.	never	mod.	mod.	mod.	mod.	heavy	never	mod.
Stroke-Related										
Type	Hem.	Ische.	Hem.	Hem.	Hem.	Ische.	Hem.	Ische.	Ische.	Ische.
Side	L	L	R	R	R	L	R	L	L	R
Lesional	Cortex	Cortex	Basal ganglia	Basal ganglia	Basal ganglia	Medulla	Basal ganglia	Medulla	Cortex	Cortex
HTD	no	no	yes	yes	yes	yes	yes	yes	no	no
DM	yes	yes	yes	yes	yes	no	no	no	yes	no
HLD	no	no	no	no	no	no	no	no	no	yes
Clinical Assessment										
FAC_onset_	1	0	1	0	0	0	0	0	2	2
FAC_final_	5	5	5	5	4	4	5	4	5	5
FMA_LE_onset_	4	6	27	21	15	21	33	10	20	29
FMA_LE_final_	34	29	33	29	30	29	33	18	29	34
Spatiotemporal Features (mean ± SD)										
Velocity (cm/s)	71.88±2.92	40.13±7.19	92.32±10.78	82.59±20.87	49.90±8.49	48.31±11.06	89.46±9.96	45.50±13.74	62.89±22.79	85.54±14.92
Cadence (steps/min)	92.11±10.96	69.51±10.17	91.78±11.09	87.71±12.07	81.99±10.45	68.55±10.65	95.47±6.18	74.11±7.13	79.59±12.15	95.16±6.14
Stride length (cm)	92.92±14.35	69.70±22.41	121.41±8.53	111.90±15.48	73.43±14.39	83.78±12.47	112.58±8.15	73.37±10.47	93.06±15.89	107.29±9.60

**Table 2:** Summary of missing data.

No. of Weeks w/o Missing	No. of Patients
8	7
7	6
6	5
5	9
4	4

**Table 3:** Hyperparameter settings for TimeVAE tuning.

Hyperparameter	Search Range	Selected Value
Epochs	10,000	10,000
Learning rate	[1 × 10^−5^, 1 × 10^−4^, 1 × 10^−3^]	1 × 10^−4^
Latent dimension	[2, 4, 8, 16]	2
Batch size	[32, 64, 128]	64
Number of layers	[2, 3, 4]	3

**Table 4:** Statistical significance of TimeVAE and Diffusion-TS augmentation.

Model	${\mathrm{MMD}}_{\mathrm{obs}}^{2}$	*p*-Value
TimeVAE	0.065	0.069
Diffusion-TS	0.228	<0.001

**Table 5:** Hyperparameter search ranges and selected values.

Hyperparameter	Search Range	Selected Value
Epochs (TAE)	150	150
Epochs (clustering)	100	100
Batch size	168 (full-batch)	168 (full-batch)
Learning rate (TAE)	(5 × 10^−3^, 1 × 10^−2^)	6.761 × 10^−3^
Learning rate (clustering)	(5 × 10^−4^, 5 × 10^−3^)	5.534 × 10^−4^
Pooling size	[2, 4, 6, 8]	6
Hidden size (Bi-LSTM 1)	50	50
Hidden size (Bi-LSTM 2)	1	1

**Table 6:** Clustering performance scores when using only raw data versus when using augmented data as well.

K	Raw			Raw + VAE		
	BSS	SS	H	BSS	SS	H
3	0.757	0.430	0.993	0.779	0.518	0.889
4	0.715	0.343	0.957	0.768	0.485	0.913
5	0.711	0.356	0.899	0.766	0.534	0.759
6	0.707	0.339	0.918	0.780	0.533	0.859
7	0.691	0.283	0.969	0.745	0.484	0.759
8	0.661	0.215	0.961	0.723	0.419	0.803
9	0.672	0.246	0.952	0.702	0.317	0.951
10	0.679	0.258	0.960	0.721	0.377	0.907

**Table 7:** Actual patients assigned to each gait recovery group.

Group	No. of Members	Actual Patient ID
A	23	8, 12, 20
B	35	10, 13, 14, 15, 22, 27, 31
C	67	1, 2, 3, 4, 7, 9, 11, 19, 24, 28, 29
D	10	6
E	25	5, 17, 18, 23
F	26	16, 21, 25, 26, 30

**Table 8:** Statistical significance of kinematic features using two-way ANOVA.

Joint	Metric	F	*p*
	Peak extension	1.723	<0.01
Hip	RMSE (stance phase)	1.888	<0.005
	RMSE (swing phase)	1.773	<0.005
	Peak flexion (swing phase)	1.621	<0.05
Knee	RMSE (stance phase)	1.405	0.060
	RMSE (swing phase)	1.254	0.150
	Peak dorsiflexion (terminal stance phase)	1.771	<0.005
Ankle	RMSE (stance phase)	1.182	0.218
	RMSE (swing phase)	1.182	<0.05

## Data Availability

All datasets have been anonymized to protect patient confidentiality. The data are not publicly available due to the regulation of SMC Institutional Review Board (SMC 2017-11-081).
